# Hepatitis B infection is associated with periodontitis: the national health and nutrition examination survey (2009–2014)

**DOI:** 10.1186/s12903-024-04489-y

**Published:** 2024-07-17

**Authors:** XianRun Chen, Jukun Song, JiangLing Sun, JiQin Zhang, XingJin Chen, ChongWen Zeng, JiaXin Hu, XingTao Chang, FuQian Jin, SiYang Luo, Zhu Chen, Yi Luo

**Affiliations:** 1 School of Stomatology, Zunyi Medical University, Zunyi, China; 2 Department of Prosthodontics, Guiyang Stomatological Hospital, Guiyang, China; 3 Department of Endodontics, Guiyang Stomatological Hospital, Guiyang, China; 4 Department of Oral and Maxillofacial Surgery, The Affiliated Stomatological Hospital of Guizhou Medical University, Guiyang, China

**Keywords:** Hepatitis B infection, Periodontitis, NHANES

## Abstract

**Background:**

Current research has been inconclusive regarding whether hepatitis B infection is associated with an increased risk of periodontitis. This study aims to test the null hypothesis that no association exists between hepatitis B infection and an increased risk of periodontitis using the National Health and Nutrition Examination Survey (2009–2014).

**Methods:**

We performed a cross-sectional study using the National Health and Nutrition Examination Survey (NHANES) database (2009–2014) to assess the rate of the prevalence of periodontitis in patients with and without hepatitis B infection. Participants who had tested for hepatitis B and periodontitis were included. The included participants were divided into no/mild periodontitis and moderate/severe periodontitis groups according to their periodontal status. The association between hepatitis B infection and chronic periodontitis was evaluated by multivariable regression analyses adjusting for age, gender, race/ethnicity, education level, income-to-poverty ratio, smoking, alcohol, BMI, ALT, AST, creatinine, hypertension, and diabetes.

**Results:**

A total of 5957 participants were included and divided into two groups: inactive periodontitis group (*n* = 3444) and active periodontitis group (*n* = 2513). The results showed that participants with hepatitis B had a higher risk of periodontitis. After adjusting for covariables, adults with hepatitis B infection were 38% more likely to have periodontitis compared to those without hepatitis B infection (95% Confidence Interval [CI]:1.085–1.754).

**Conclusions:**

In general, the results suggest that CHB is positively associated with the more severe periodontitis. These results suggest that people with hepatitis B infection should take good periodontal care measures to avoid the occurrence and development of periodontitis.

## Introduction

The burden of periodontitis continues to be a global public health problem, with the majority of people with periodontitis aged between 55 and 59 years; however, the prevalence of periodontitis is increasing in younger people [1]. There are more than 700 types of bacteria in the oral cavity, and these bacteria usually co-exist in a harmonious state, called probiotics, when symbiotic bacteria do not allow harmful bacteria to cause disease [2]. These bacteria can also be found in the gingival groove or sulcus, the narrow space that is delimited by the tooth’s surface and the gingiva [3]. If the gingival sulcus is not cleaned properly and regularly through professional and at-home methods, this can lead to the emergence of highly pathogenic bacteria. Therefore, this subgingival pathogenic biofilm causes periodontal inflammation (periodontitis) [4]. Periodontitis is a chronic inflammation that damages the supporting tissues of the teeth and, if the condition continues to develop, can lead to loss of alveolar bone, loosening of teeth, and eventually tooth loss [5]. In the past few decades, a large number of studies have shown that the consequences of periodontitis are not only the destruction of normal tooth function and structure [6]. Periodontal tissue is connected to the rest of the body by blood and lymph [7]. Therefore, every pathological change that can disrupt general homeostasis has the potential to affect periodontal health. At the same time, periodontitis can also affect the overall health of patients, as well as the occurrence and development of specific diseases [8]. Researchers studied the bi-directional relationship between periodontitis and systemic health and disease, resulting in the concept of “periodontal medicine” [9]. More and more literature studies show that periodontitis is associated with many systemic diseases, such as diabetes, Alzheimer’s disease, and respiratory infection. The biological mechanism underlying this correlation still needs further study [10].

The oral cavity is a dynamic open system with various ecological niches in various forms and conditions, which is a suitable environment for microbial colonization [11, 12]. In addition to being colonized by bacteria, archaea, and fungi, the mouth can harbor a variety of viruses. Cytomegalovirus, Epstein-Barr virus, human herpesvirus 6, HIV, and herpes simplex virus type 1, as well as hepatitis A, B, and C, anelloviruses (such as torque teno virus), and papillomaviruses, can be detected in saliva and gingival tissues [13]. In a recent meta-analysis, several of these viruses were found to be associated with periodontitis [14], possibly through direct damage, coordination with the local bacterial community, and/or regulation of the immune response [15].

Hepatitis B is an infection caused by hepatitis B virus that damages the liver [16]. The virus is mainly transmitted through contact with infected blood or bodily fluids [17]. The infection enters a chronic phase, which can lead to life-threatening complications such as cirrhosis and liver cancer [18]. A meta-analysis showed that there is sufficient evidence in the existing literature to support the effect of HBV infection on the oral environment [19]. Studies on the effects of hepatitis B virus infection on the oral cavity mainly involve the detection of viral antigens in saliva and gingival crevicular fluid and rarely involve the clinical, dental, or periodontal conditions of patients [19].

Furthermore, previous research has shown a correlation between periodontitis and liver disease [20, 21]. A retrospective study from Japan suggests that periodontitis may be associated with the progression of viral liver disease [20]. Impaired oral health in patients with hepatitis C infection can be the result of liver dysfunction, compromised immune systems, or a lack of motivation for infected patients to seek dental care [22, 23]. A study utilizing Escherichia coli lipopolysaccharide and Streptomyces griseus proteases to induce periodontitis and steatosis in an animal model demonstrated this relationship [24]. Additionally, Porphyromonas gingivalis, a significant periodontal pathogen, has been found in the livers of patients with hepatic fibrosis, impacting the progression of liver disease [25].

Although the relationship between periodontitis and other liver diseases has been analyzed and evaluated from different perspectives [20–25], the relationship between periodontitis and hepatitis B needs to be further studied. It is valuable to study the relationship between HBV infection and periodontitis in a large representative population. Therefore, we analyzed secondary data based on available data from the National Health and Nutrition Examination Survey (NHANES). The study aims to determine whether there was a significant relationship between hepatitis B infection and periodontitis and to understand the associated confounding factors. Therefore, this study tests the null hypothesis that there is no difference in the prevalence of periodontitis between individuals with and without hepatitis B infection.

## Materials and methods

### Study design and data source

The NHANES is a continuous survey research that provides population estimates related to the nutrition and health of adults and children in America. The survey employs a stratified, multistage probability design to recruit a representative sample of the American population. Data were obtained via personal structured interviews at home, health examinations at a mobile examination center, and specimen analyses in the laboratory. NHANES 2009–2014 was approved by The National Center for Health Statistics (NCHS) Ethics Review Board and conducted following the Helsinki Declaration of 1975, as revised in 2013. Informed consent was obtained from all participants. The acquisition and analysis of data was consistent with NHANES research requirements.

In this cross-sectional retrospective study, we selected NHANES data from the 2009–2010, 2011–2012, and 2013–2014 cycles. The Centers for Disease Control and Prevention (CDC) manages the National Center for Health Statistics (NCHS), which conducts NHANES assessments of the health and nutrition status of children, adults, and older adults. All NHANES protocols were approved by the National Center for Health Statistics Ethics Review Committee with written informed consent from all participants [26]. This modeling investigation was exempt from review because it used published de-identified data sets that included no personally identifiable information. The data from the CDC website can be downloaded for free (https://wwwn.cdc.gov/nchs/nhanes/Default.aspx).

**Fig. 1:**
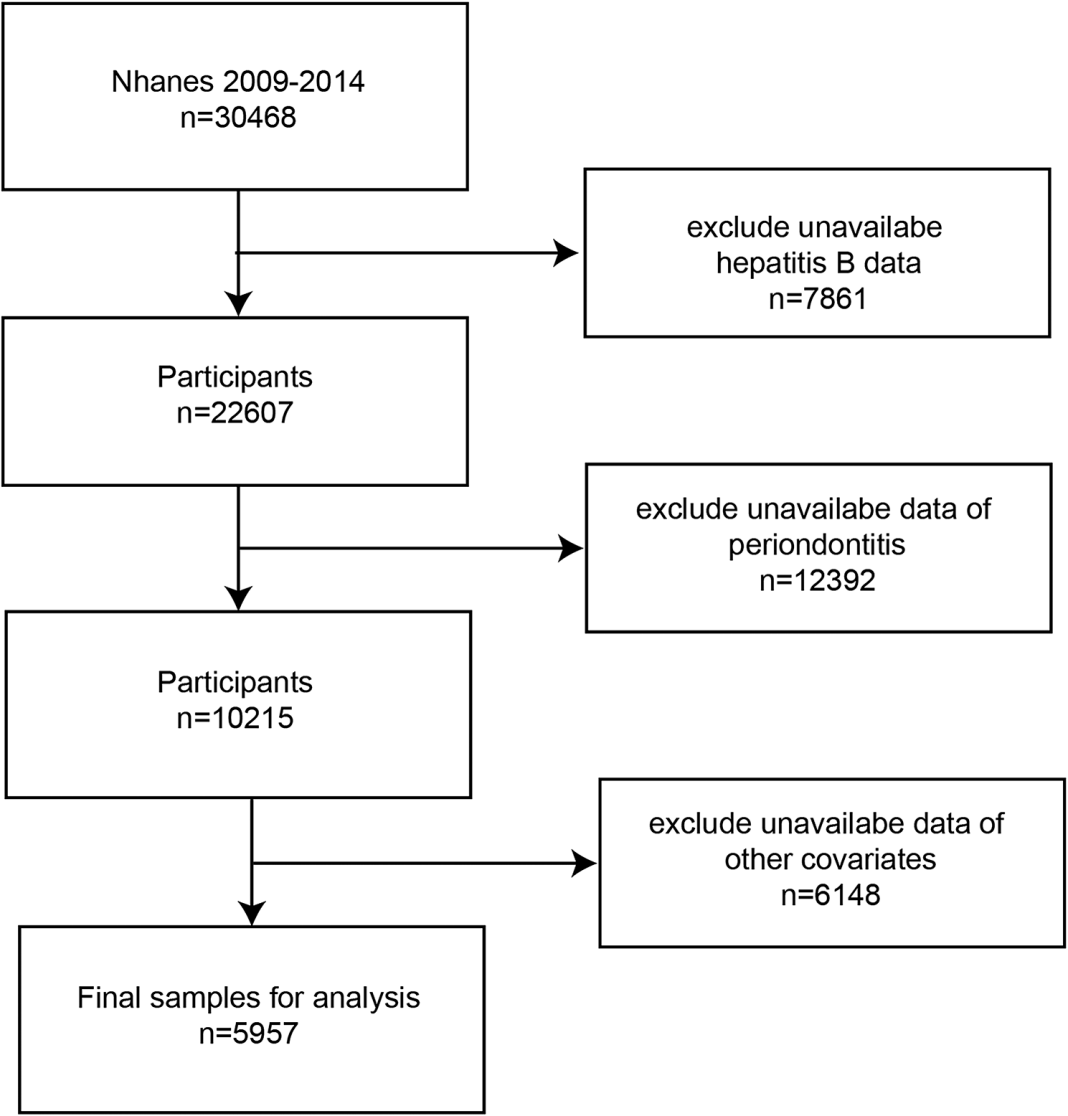
Flowchart of the study design. *NHANES* National Health and Nutrition Examination survey

The Inclusion and exclusion procession is shown in Fig. 1. We used the data from the NHANES 2009–2014 cycles. 5957 patients with clear hepatitis B, periodontitis, and other covariable data were included to explore the association between chronic hepatitis B infection and the risk of periodontitis. Among them, 419 had hepatitis B infection, while 5,538 had never had hepatitis B infection.

### Assessment of periodontitis

The dependent variable was periodontitis (dichotomous variable). A mobile examination center (MEC) was used for periodontal examination of participants aged 30 years and older. For oral health examinations, the NHANES operating manual describes the training and calibration process [27]. According to the 2012 CDC/ American Association of Periodontitis (AAP) case definition for periodontitis, participants must have at least two teeth that meet specific detection thresholds, and there are four grades of periodontitis: no, mild, moderate, and severe [28]. Mild periodontitis was defined as ≥ 2 interproximal sites with AL ≥ 3 mm and ≥ 2 interproximal sites with PD ≥ 4 mm (not on the same tooth) or one site with PD ≥ 5 mm. Moderate periodontitis was defined as ≥ 2 interproximal sites with AL ≥ 4 mm (not on the same tooth), or ≥ 2 interproximal sites with PD ≥ 5 mm (not on the same tooth). Severe periodontitis was defined as ≥ 2 interproximal sites with AL ≥ 6 mm (not on the same tooth) and ≥ 1 interproximal site with PD ≥ 5 mm. This study defines people with no and mild periodontitis as inactive periodontitis, and people with moderate and severe periodontitis as active periodontitis.

### HBV serological testing

In this study, Anti-HBc was used as a key indicator because hepatitis B surface antigen positive indicates that the patient is currently infected with acute or chronic hepatitis, but does not include clinically cured cases [29]. However, HBV core antibodies remain positive after the acute phase of inflammation has resolved, so the selection of HBV core antibody-positive patients can include all patients with current or previous hepatitis B infection [30]. Hepatitis B core antibodies are tested by using the VITROS Anti-HBc test. The VITROS Anti-HBc assay is performed using the VITROS Anti-HBc Reagent Pack and VITROS Immunodiagnostic Products Anti-HBc Calibrator on the VITROS ECi/EciQ Immunodiagnostic System.

### Assessment of covariates

In the demographic data in the NHANES database, we collected relevant information about the respondents’ age, gender, race, income level, education, etc. Ethnicity is divided into Mexican Americans, other Hispanics, non-Hispanic whites, non-Hispanic blacks, and others. The family income level is divided into low-income, middle-income, and high-income. The level of education is classified as below high school, high school or equivalent, university or above, and others.

The examination information contains the physical examination data of the respondents. In this column, we collected data on the body mass index (BMI) of the respondents. BMI is calculated based on a person’s weight and height(<18.5 kg/m^2^,18.5–24.9 kg/m^2^, 25.0–29.9 kg/m^2^, > = 30.0 kg/m^2^) [31].

In the Laboratory test data, the correlation results of the respondent tests are recorded. Here, we collected data on hepatitis B core antibodies (anti-HBc). In addition, data were collected for relevant covariates ALT, AST, and creatinine that may have influenced the results.

Finally, we collected data about smoking, alcohol, hypertension, and the presence of diabetes in the respondents in the questionnaire column. Smoking status was categorized as “nonsmokers” (lifetime use of < 100cigarettes), “former smokers” (previous history of smoking but no longer a smoker at the time of interview), or “current smokers” (lifetime use of ≥ 100 cigarettes and who currently smoke cigarettes) [32]. According to the average number of alcoholic drinks consumed per week in the past 12 months, alcohol consumption was categorized into three strata (0–1, 1-< 8, and ≥ 8 drinks per week) and defined as nonuse, moderate use, and heavy use, respectively. A “drink” was defined as a 12-ounce beer, a 5-ounce glass of wine, or one-and-half ounces of liquor [33].

### Statistical analyses

According to the hepatitis indicators, the respondents who were positive and negative for hepatitis were divided into two different groups. In terms of data statistics, the data of categorical variables are expressed as numbers with percentages (*N*%), while the data of continuous variables are expressed as mean values with standard deviations (mean ± SD). We used Chi-square tests to compare the percentages of categorical variables between the different groups. Multivariable Logistic regressions were used to examine the association between hepatitis B infection and periodontitis. We used the null hypothesis that there was no relationship between hepatitis B infection and periodontitis. The crude model does not adjust for covariates, Model I adjusts for sociodemographic data, and Model II adjusts for all covariates. Odds ratios (ORs) were calculated with 95% confidence intervals (CIs) from multivariate logistic regression analyses were reported to demonstrate the observed associations. At the same time, we stratified the relationship between hepatitis B infection and periodontitis by gender and BMI to assess whether there is a potential impact change between hepatitis B infection and periodontitis. All the analyses were performed with the statistical software packages R (http://www.R-project.org, The R Foundation) and Empower Stats (http://www.empowerstats.com, X&Y Solutions, Inc, Boston, MA). All tests were two-sided and P values lower than 0.05 were considered statistically significant, and the null hypothesis was rejected.

## Results

### Description of the study sample

The study sample consisted of 5957 participants from 2009 to 2014. Approximately 7.0% of participants had hepatitis B infection, and 42.18% suffered from periodontitis. The subjects in the group with active periodontitis are more likely to be 55–69 years old, male, non-Hispanic white, with higher BMI, less smoking (less than 100 cigarettes), no hypertension and diabetes, and moderate use of alcohol (*p* < 0.05) (Table 1). In Table 2, we show the periodontal clinical indicators of hepatitis B-infected and uninfected patients. The results showed that the average Pocket depth and the average degree of attachment loss of hepatitis B infected people were deeper and more serious than that of uninfected people, and more sites with PD ≥ 4 mm and AL ≥ 3 mm. In addition, the proportion of active periodontitis is also higher in the hepatitis B-infected population.

**Table 1:** Baseline characteristics of participants in the NHANES

Periodontitis	Inactive	Active	*p*-value
**N**	3444	2513	
**ALT**	26.25 ± 18.65	27.70 ± 22.96	0.006
**AST**	26.28 ± 24.19	27.62 ± 18.51	< 0.001
**Creatinine**	76.96 ± 21.68	82.14 ± 26.41	< 0.001
**Hepatitis B**			< 0.001
No	3272 (95.01%)	2266 (90.17%)	
Yes	172 (4.99%)	247 (9.83%)	
**Gender**			< 0.001
Male	1579 (45.85%)	1668 (66.37%)	
Female	1865 (54.15%)	845 (33.63%)	
**Age**			< 0.001
30–44	1752 (50.87%)	624 (24.83%)	
45–54	771 (22.39%)	594 (23.64%)	
55–69	672 (19.51%)	900 (35.81%)	
> 69	249 (7.23%)	395 (15.72%)	
**Race**			< 0.001
Mexican American	352 (10.22%)	424 (16.87%)	
Other Hispanic	313 (9.09%)	216 (8.60%)	
Non-Hispanic White	1911 (55.49%)	1057 (42.06%)	
Non-Hispanic Black	508 (14.75%)	600 (23.88%)	
Other	360 (10.45%)	216 (8.60%)	
**Education**			< 0.001
Less than HS	423 (12.28%)	672 (26.74%)	
HS graduate	597 (17.33%)	651 (25.91%)	
Some coll./tech.	1047 (30.40%)	708 (28.17%)	
Coll./tech. graduate	1377 (39.98%)	482 (19.18%)	
**PIR**			< 0.001
<2.00	1065 (30.92%)	1221 (48.59%)	
≥ 2.00	2379 (69.08%)	1292 (51.41%)	
**Smoking status**			< 0.001
Never	530 (15.39%)	755 (30.04%)	
Former	853 (24.77%)	778 (30.96%)	
Current	2061 (59.84%)	980 (39.00%)	
**Alcohol consumption**			< 0.001
Nonuse	1356 (39.37%)	888 (35.34%)	
Moderate use	1819 (52.82%)	1288 (51.25%)	
Heavy use	269 (7.81%)	337 (13.41%)	
**BMI**			0.004
<18.5	21 (0.61%)	34 (1.35%)	
18.5–24.9	931 (27.03%)	617 (24.55%)	
25-29.9	1227 (35.63%)	895 (35.61%)	
≥30	1265 (36.73%)	967 (38.48%)	
**Hypertension**			< 0.001
Yes	1040 (30.20%)	1058 (42.10%)	
No	2404 (69.80%)	1455 (57.90%)	
**Diabetes**			< 0.001
Yes	245 (7.11%)	348 (13.85%)	
No	3199 (92.89%)	2165 (86.15%)	

Abbreviations: ALT: Alanine aminotransferase; AST: Aspartate aminotransferase; PIR: poverty income ratio. BMI: body mass index

**Table 2:** Periodontal clinical indexes of HBV infected and uninfected patients

Hepatitis B	Negative	Positive	*p*-value
N	5538	419	
Mean PD (mm)	1.517 ± 0.627	1.606 ± 0.694	0.002
Mean AL (mm)	1.734 ± 1.101	2.131 ± 1.304	< 0.001
Percentages of sites with PD ≥ 4 mm (%)	2.790 ± 6.963	3.395 ± 6.809	< 0.001
Percentages of sites with AL ≥ 3 mm (%)	13.563 ± 16.332	18.569 ± 17.421	< 0.001
Peridontitis			< 0.001
Inactive	3272 (59.083%)	172 (41.050%)	
Active	2266 (40.917%)	247 (58.950%)	

Abbreviations: PD: Pocket depth; AL: Attachment loss

### Relationship between hepatitis B infection and periodontitis

The multivariable logistic regression analysis between chronic hepatitis B and periodontitis is shown in Table 3. The positive association between hepatitis B and periodontitis was revealed in all models. Model I only adjusts for sociodemographic data, and the adjusted OR increases relatively (OR 1.390, 95% CI 1.100–1.756, *p* = 0.00574). After adjusting for all covariates in Model II, the OR value does not change much compared to Model I(OR 1.380, 95% CI 1.085–1.754, *p* = 0.00857). We stratified the analysis by gender and BMI (Table 4). The results showed that among those infected with hepatitis B, males and those with a high BMI had a higher risk of developing periodontitis.

**Table 3:** Association between hepatitis B infection and periodontitis in multivariable logistic regression

	OR (95%CI), *p*-value		
	Crude model	Model I	Model II
**Hepatitis B**			
Negative	1.0	1.0	1.0
Positive	2.074 (1.695, 2.537) < 0.00001	1.390 (1.100, 1.756) 0.00574	1.380 (1.085, 1.754) 0.00857

Abbreviations: CI: confidence interval; OR: odds ratio

The crude model adjusts for: None

Model I adjusts for sex; age; race; education level; and PIR.

Model II adjust for: sex; age; race; education level; PIR; BMI; ALT; AST; creatinine; smoking; alcohol consumption; hypertension; history of diabetes;

**Table 4:** Stratified analyses of periodontitis in respondents, according to gender and BMI.

Hepatitis B			
	**N**	**Active periodontitis**	*p*-value
**Gender**			
Male	3247	2.440 (1.768, 3.367)	< 0.0001
Female	2710	1.252 (0.892, 1.757)	0.1948
**BMI**			
<18.5	55	inf. (0.000, Inf)	0.9947
18.5–24.9	1548	1.681 (1.099, 2.572)	0.0166
25-29.9	2122	1.470 (1.013, 2.135)	0.0428
≥30	2232	2.050 (1.376, 3.055)	0.0004

Abbreviations: BMI: body mass index

## Discussion

We conducted a cross-sectional study of the U.S. population using data from the NHANES database. We included a total of 5,957 participants and showed a positive association between hepatitis B infection and periodontitis. This positive association persisted after adjusting for covariates (sex, age, race, education, PIR, smoking, alcohol consumption, BMI, AST, ALT, creatinine, hypertension, and diabetes). The adjusted odds ratio of hepatitis B to periodontitis suggests that hepatitis B infection is a risk factor for the development of periodontitis.

In some previous studies, the potential mechanism by which hepatitis B infection affects periodontitis was discussed. Studies conducted between 1984 and 1985 focused on detecting HBV antigens (or viral particles) in the oral fluid of infected patients. Periodontal tissue is connected to the rest of the body by blood and lymph [7]. Viral particles circulate in the blood, reach the lymphatic system, and end up in the GCF due to differences in osmotic pressure. GCF is then used as a carrier to reach the saliva [34]. This hypothesis is further supported by a recent study by Kamimura et al., who concluded that the detection of hepatitis B virus DNA is positively correlated with the detection of hepatitis B virus in salivary occult blood [35]. In addition, Amir et al. tested the levels of ALT and AST in women with chronic periodontitis and found a correlation between periodontal index and serum liver enzyme levels [36]. Concurrently, AST and ALT serve as sensitive markers for liver damage and are also indicative of the progression of viral hepatitis [37].

From an immunological perspective, enzyme-linked immunosorbent assay (ELISA) performed on saliva samples from HBV-infected patients showed significantly increased levels of pro-inflammatory interleukin (IL-2 and IL-4) and anti-inflammatory interleukin (IL-10) in saliva samples from HBV-infected patients compared to healthy controls [38]. Gharavi et al. also showed that the same immunoassay (ELISA) has good sensitivity and specificity for the diagnosis of HBV infection in saliva samples [39]. Thus, an imbalance in inflammatory mediators may support a bidirectional relationship between hepatitis B infection and periodontitis.

The conclusion of the retrospective study by Nagao et al. also highlights that periodontitis may be associated with the progression of viral liver disease; Therefore, the control of oral diseases is essential for the prevention and management of liver fibrosis [20]. They found obesity as a risk factor for periodontal disease among patients with viral liver disease [20]. This is consistent with the results of our stratified analysis. With the increase in BMI, the risk of periodontitis in hepatitis B patients increased significantly. The meta-analysis showed a significant association between obesity and periodontitis (OR = 1.30 [95% Confidence Interval (CI), 1.25–1.35]) and with mean Body Mass Index (BMI) and periodontal disease (mean difference = 2.75) [40]. Obesity is also a key component of metabolic syndrome and is associated with an increased relative risk of cancer in many tissues, including the liver [41]. Concurrently, obesity, diabetes, and metabolic syndrome have the potential to expedite the advancement of liver disease in individuals with chronic hepatitis B virus infection, ultimately leading to the onset of cirrhosis and potentially hepatocellular carcinoma [42]. In addition, periodontitis is also associated with metabolic syndrome [43].

In the clinical study of the relationship between liver disease and oral disease. The compromised periodontal health observed in individuals with hepatitis C infection may stem from liver dysfunction, weakened immune responses, or a diminished inclination among infected patients to pursue dental treatment [22, 23]. Numerous scholarly investigations have examined the impact of HCV infection on the oral cavity, emphasizing the dental pathological alterations and additional extrahepatic manifestations (EHMs) that have implications for oral health [44]. Yang et al. conducted a prospective study on the link between tooth loss and the incidence of primary liver cancer, showing that an increased number of lost teeth is associated with a higher risk of primary liver cancer [45]. The study by Dong-hun Han et al. also found that viral hepatitis may be associated with methyl mercaptan-defined halitosis [46]. We have reason to think that liver disease may harm the oral environment.

We used the NHANES database from the United States. Due to the large sample size of the data, it can well represent the adult respondents related to chronic hepatitis B infection and periodontitis in the United States. Compared with previous clinical studies, our research results are more objective and representative to a certain extent. In a previous meta-analysis, it was noted that HBV infection and its oral effects mainly involved the detection of viral antigens in saliva and gingival fluid, and were less involved in the clinical, dental, or periodontal status of infected patients [19]. Therefore, our cross-sectional study makes up for this deficiency to some extent and is an important supplement to the existing knowledge base.

However, despite these advantages, our study still has some limitations. First, because this is a cross-sectional study, cause-and-effect relationships cannot be confirmed. Second, due to the limited research on hepatitis B and periodontitis, the scope of discussion is limited. We are unable to explain the higher risk of periodontitis in men with hepatitis B in the study, and the mechanism needs to be further studied. Moreover, this is even though the 2012 AAP/CDC case definition of periodontitis has been used in the past as a global standard for epidemiological studies of periodontal disease [47]. In 2018, the European Federation of Periodontology/American Academy of Periodontology (EFP/AAP) published a new classification of periodontitis and called for it to replace the 2012 CDC/AAP Case Definition of periodontitis [48, 49]. CAL is recognized as the gold standard for periodontitis severity and progression, but it may be difficult to distinguish between incipient periodontitis and gingivitis when used alone [47]. However, the cause of CAL has not been documented in the NHANES periodontitis database. Therefore, using the 2018 EFP/AAP classification of periodontal diseases to analyze NHANES periodontitis data may lead to an increase in the prevalence of periodontitis [50, 51]. CAL and PD were used in the 2012 AAP/ CDC case definition of periodontitis to minimize the potential erroneous influence of periodontal retreat on the results of probing depth measurements [47]. In addition, due to the missing data of some variables in the NHANES database, in order to ensure the robustness of the results, we included all covariables and cleaned the data, leaving only 419 hepatitis B infection positive samples. The sample size of HBV positive is relatively small, which may affect the analysis results. Finally, because this is a large national survey, there may be confounding factors that could affect our results due to measurement errors and unmeasured variables. Therefore, this study cannot reflect the relationship between hepatitis B infection and periodontitis, so more relevant variables should be considered in the future to conduct longitudinal studies.

## Conclusions

This cross-sectional study suggested that hepatitis B infection is a risk factor for periodontitis. Individuals infected with hepatitis B may be at an increased risk for periodontal diseases, necessitating enhanced oral health surveillance and care. Our study suggests that special attention should be given to men and obese individuals, who may be particularly vulnerable to severe periodontal conditions. Regular oral health examinations and proactive periodontal interventions could potentially mitigate these risks. The pathophysiological mechanism needs further study.

## Data Availability

Publicly available datasets were analyzed in this study. This data can be found here: https://wwwn.cdc.gov/nchs/nhanes/.

## Abbreviations

NHANES: The national health and nutrition examination survey
HBV: Hepatitis B virus
ALT: Alanine aminotransferase
AST: Aspartate aminotransferase
PIR: Poverty income ratio
BMI: Body mass index
CDC: The Centers for Disease Control and Prevention
NCHS: The national center for health statistics
AAP: American Association of Periodontitis
CI: Confidence interval
OR: Odds ratio
AL: Attachment loss
PD: Pocket depth
